# Comparison of efficacy between coaxial microincision and standard-incision phacoemulsification in patients with age-related cataracts: a meta-analysis

**DOI:** 10.1186/s12886-017-0661-6

**Published:** 2017-12-29

**Authors:** Lijun Wang, Xiao Xiao, Lin Zhao, Yi Zhang, Jianming Wang, Aiyi Zhou, Jianchao Wang, Qian Wu

**Affiliations:** 1 Department of Ophthalmology, the Second Affiliated Hospital of Xi’an Jiaotong University, 157 Xiwu Road, Xi’an, 710004 China; 2 Department of Ophthalmology, the Central Hospital of Shaanxi Xi’an, 161 Xiwu Road, Xi’an, 710004 China; 3 School of Public Health, Xi’an Jiaotong University Health Science Center, 76 West Yanta Road, Xi’an, 710061 China

**Keywords:** Microincision, Standard incision, Age-related cataract, Phacoemulsification, Meta-analysis

## Abstract

**Background:**

Incision size plays a critical role in the efficacy of cataract surgery, but the available evidence on ideal incision size is inconsistent. In this study, we conducted a meta-analysis to evaluate the efficacy of coaxial microincisional phacoemulsification surgery (MICS) compared with that of standard-incision phacoemulsification surgery (SICS) in patients with age-related cataracts.

**Methods:**

The Cochrane Library (Wiley Online Library), PubMed, Medline, National Knowledge Infrastructure (CNKI), and VIP databases were searched to identify reports of clinical randomized controlled trials (RCTs) comparing MICS to SICS for the treatment of age-related cataracts. The outcomes of interest included surgically induced astigmatism (SIA), effective phacoemulsification time (EPT), central corneal thickness (CCT), endothelial cell count (ECC), endothelial cell count loss (ECC Loss %), and average ultrasonic energy (AVE).

**Results:**

Eleven RCT studies were included in this meta-analysis. No statistically significant differences were observed in EPT (Z = 1.29, *P* > 0.05), CCT (1 day: Z = 1.37, P > 0.05; 7 days: Z = 0.75, P > 0.05; 30 days: Z = 0.38, P > 0.05; 90 days: Z = 0.29, P > 0.05), ECC (7 days: Z = 1.13, P > 0.05; 30 days: Z = 1.42, P > 0.05) or ECC Loss % (7 days: Z = 0.24, P > 0.05; 30 days: Z = 0.06, P > 0.05; 90 days: Z = 0.10, P > 0.05) between MICS and SICS. However, statistically significant differences were found in AVE (Z = 4.19, *P* < 0.0001) and SIA (1 day: Z = 10.33, *P* < 0.00001; 7 days: Z = 10.71, P < 0.00001; 30 days: Z = 10.95, P < 0.00001; 90 days: Z = 2.21,- *P* < 0.01) between MICS and SICS.

**Conclusion:**

Compared with SICS, MICS can reduce short-term and long-term SIA, but it does not differ in safety outcomes or in the time required for surgery.

**Electronic supplementary material:**

The online version of this article (10.1186/s12886-017-0661-6) contains supplementary material, which is available to authorized users.

## Background

Age-related cataracts are a common condition and one of the most important causes of blindness. With the population increasing at a rate of more than 10 million people per year and as life expectancy continues to rise, 0.4 to 1.2 million new cataract patients are expected every year in China, and the number of cataract-related blindness cases is expected to increase to 5.0625 million in 2020 [1]. Due to improvements in medical technology and surgical instruments, phacoemulsification has now become a mainstream treatment for cataracts. Cataract surgery has gradually evolved from blindness prevention surgery to refractive surgery, with the aim of not only restoring vision but also improving visual quality and quality of life. The choice of surgical incision plays a crucial role in the efficacy of surgery, as the incision damages the surrounding tissues and affects the surgical approach. The size of microincisional phacoemulsification surgery (MICS) incisions ranges from 1.8 mm to 2.2 mm, whereas standard-incision phacoemulsification surgery (SICS) incisions range from 2.8 mm to 3.2 mm [2]. As the field of cataract surgery has trended towards minimally invasive approaches in recent years, some scholars now hold the view that smaller incisions contribute to less surgically induced astigmatism (SIA) [3–5] and hasten healing of the incision, thus leading to faster post-surgical recovery [6–8]. However, smaller incisions require high technical proficiency on the part of the surgeon, as well as sophisticated surgical instruments. Smaller incisions increase the difficulty of surgery and influence the outcomes, as they limit the range of movement of the surgical instruments. Research results on the comparative efficacy of MICS and SICS in patients with age-related cataracts are inconsistent [9–11]; while some scholars suggest that MICS can effectively reduce SIA in both the short term and the long term compared with SICS [11], other studies indicate no significant difference between MICS and SICS with regard to long-term SIA [9]. Which has more advantages? MICS or SICS? Therefore, in this study, we used meta-analysis methods to examine the advantages and disadvantages by comparing the efficacy of MICS and SICS.

## Methods

### Materials

We collected all existing reports of clinical randomized controlled trials (RCTs) on MICS and SICS for the treatment of age-related cataracts published through January 2016.

### Search strategy

We searched the Cochrane Library (Wiley Online Library, 1999), PubMed, Medline, National Knowledge Infrastructure (CNKI), and VIP electronic databases. The databases were searched in October 2015 and an update was finished at January 2016 without restricting the publication status, year, language, or methodology. The search strategy combined terms related to disease (cataract) with terms related to therapies (phacoemulsification, microincisional, and standard incision). The following search strategy was used: (“cataract” OR “age related cataract” OR “senile cataract”) AND (“phacoemulsification” OR “ultrasonic emulsification for cataract”) AND (“micro incision” OR “MICS” OR “standard incision” OR “SICS” OR “Incision”). The details could be referenced to Additional file 1: Table S1. Once relevant articles were identified, their references were searched as additional articles. All the studies included in this meta-analysis were searched either from the databases or from references of relevant articles. The assessment of search results were conducted by two evaluators (W.L.J, Z.Y) independently. If the evaluators’ opinions differed, they attempted to reach a consensus and requested help from the study supervisor (W.Q). If a study was considered relevant, the full-text of the article was reviewed.

### Inclusion criteria

- Study type: randomized controlled trials (RCTs);
- Population: patients with age-related cataracts;
- Intervention: microincisional phacoemulsification surgery (MICS) versus standard-incision phacoemulsification surgery (SICS), studies with no difference in the surgical process between MICS and SICS, aside from the difference in incision size, and with a clear corneal incision at 9 ~ 12 clock;
- Outcomes variables: at least one of the outcomes of interest mentioned below. The outcomes were measured, at least, at one of the time points (preoperatively, intraoperatively, 1 day postoperatively, 7 days postoperatively, 30 days postoperatively, or 90 days postoperatively).

### Exclusion criteria

- studies with incomplete data and information;
- studies of patients with other ocular pathology, such as diabetes, glaucoma, corneal scars, lens dislocation, age-related macular degeneration, history of eye surgery, etc.;
- duplicate reports;
- conference abstracts;
- literature reviews;
- non-clinical experiments or animal studies;
- studies in which the surgical incision was performed on the astigmatism axis.

### Outcomes measures

The following outcomes were used to compare the efficacy between MICS and SICS.

The primary outcome is surgically induced astigmatism (SIA), which is an important factor to evaluate the efficacy of phacoemulsification. The less the astigmatism, the better the visual quality. The astigmatism was measured by corneal topography at preoperatively, 1 day postoperatively, 7 days postoperatively, 30 days postoperatively, and 90 days postoperatively.

The secondary outcomes are as follows: 1) The effective phacoemulsification time (EPT) and the average ultrasonic energy (AVE) were recorded from intraoperative phacoemulsification parameters. 2) The central corneal thickness (CCT) was measured by corneal topography at preoperatively, 1 day postoperatively, 7 days postoperatively, 30 days postoperatively, and 90 days postoperatively. 3)The endothelial cell count (ECC) was measured by specular microscopy at preoperatively, 1 day postoperatively, 7 days postoperatively, 30 days postoperatively, and 90 days postoperatively. The endothelial cell count loss (ECC Loss %), defined as the percentage of endothelial cell count reduced from baseline, was calculated on the difference of preoperative and postoperative endothelial cell count on specular microscopy. 4) The incidence of intraoperative and postoperative complications.

### Statistical analysis

RevMan software (Version 5.2, The Nordic Cochrane Centre, The Cochrane Collaboration, Copenhagen, Denmark) was used for the meta-analysis. The outcomes were extracted from the included studies to test the merged effect. The means and standard deviations of continuous outcomes were used to calculate the weighted mean difference (WMD) with a 95% confidence interval (95%CI). Whereas, odds ratios (ORs) with a 95% confidence intervals (95% CIs) were calculated for all dichotomous outcomes. The statistic significance level was set at a *P*-value less than 0.05. According to the Cochrane Handbook, the potential statistical heterogeneity was assessed using the χ^2^ test. I^2^ index score was used to describe the percentage of variability of heterogeneity and to decide whether to use a fixed or random effects model in the meta-analysis. The statistic significance level was set at a P-value less than 0.10 and an I^2^ score greater than 50%. If no significant heterogeneity was detected among the included studies (*P* ≥ 0.10, I^2^ < 50%), a fixed-effects model was selected for the remaining analyses. Instead, if significant heterogeneity was present among the included studies (*P* < 0.10, I^2^ > 50%), a random-effects model was used.

### Quality assessment criteria

The Cochrane risk and bias assessment tool was used to assess the quality of included studies. The quality assessment involved seven components (random sequence generation, allocation concealment, blinding of participants and personnel, blinding of outcome assessment, incomplete outcome data, selective reporting and other sources of bias). For each components, “yes” indicated a low risk of bias, “no” indicated a high risk of bias, and “unclear” indicated an unclear or unknown risk of bias. The quality assessment was conducted by two evaluators (W.L.J, Z.Y). If the evaluators’ opinions differed, they attempted to reach a consensus and requested help from the study supervisor (W.Q). The information of random method and follow-up was listed in Table 1. The details of risk of bias assessment on each study were listed in Table 2.

**Table 1:** Characteristics description of included RCT studies

Author	Year	Country	Type of study	Randomize Method	Design Center	Age (MICS / SICS, y)	Sex (MICS / SICS; M/F)	Source of cases	No. of eyes (MICS / SICS)	Follow-up(d)	loss to follow-up	Index	Intraoperative complications	Postoperative complications
1 LAN Jianqing	2013	China	RCT	random number	1	68.5 ± 6.4/	11/12; 7/9	H	23/16	90	11/48	a,b,c,d, e, f	CW: MICS(5/23,21.7%),SICS(5/16,2.31.2%)	NC
						68.0 ± 7.8								
2 TAN Nian	2012	China	RCT	random number	1	NA	13/15; 20/12	H	28/32	30	NA	a,b,c,e	NC	CE: MICS(5/28,17,9%),SICS(4/32,12.5%)
3 ZHANG Jianzhu	2014	China	RCT	NA	1	67.5/69.8	38/46; 44/40	H	84/84	30	0/168	c	NC	NC
4 CHEN Yongjun	2012	China	RCT	registration order	1	65.40 ± 8.72/	20/25; 22/23	H	45/45	30	0/90	c,d, e	NA	NA
						65.67 ± 8.34								
5 QIN Xufang	2014	China	RCT	NA	1	63.2 ± 1.7/	65/35; 67/33	H	100/100	7	NA	a,b,c,e	ACC: MICS(4/100,4%),SICS(3/100,3%);CW: MICS(23/100,23%),SICS(25/100,25%)	NA
						62.7 ± 1.5								
6 LI Baojiang	2014	China	RCT	NA	1	66.5/69.2	24/18; 28/14	H	42/42	30	NA	a,b,c, e, f	NC	CE: MICS(7/42,16.7%),SICS(7/42,16.7%
7 YAO Ke	2011	China	RCT	random number	1	72 ± 7	29/51	H	40/40	90	9/89	a,b,c,d, e, f	NA	NA
8 IZZET Can	2009	Turkey	RCT	NA	1	65.8 ± 13.2/	17/14; 19/14	H	45/45	90	NA	a,b,d	PCR: MICS(0/45,0%),SICS(1/45,2.2%);	NC
						66.2 ± 12.6							IPTI: MICS(1/45,2.2%), SICS(0/45,0%)	
9 JUN Wang	2009	China	RCT	NA	1	69 ± 9/71 ± 8	14/29; 14/29	H	43/44	90	NA	c,d	NC	NC
10 KEN Hayashi	2009	Japan	RCT	random number	1	70.1 ± 6.9	21/39; 21/39	H	60/60	90	0/120	c,f	NA	NA
11 LIXIAO Luo	2011	China	RCT	random number	1	73.95 ± 6.05/	21/19; 19/21	H	40/40	90	0/80	f	NC	NC
						72.48 ± 6.15								

a = EPT; b = APT; c = SIA; d = CCT; e = ECC; f = ECC Loss %; NA = not available; H = hospital; CW = corneal wrinkle; NC = no complications; CE = corneal edema; ACC = anterior chamber collapse; PCR = posterior capsule rupture, IPTI = iris prolapsed through the incision

**Table 2:** Description of bias assessment

Trial(Author)	Random sequence generation	Allocation concealment	Blinding of participants and personnel	Blinding of outcome assessment	Adequate assessment of outcomes	Selective reporting avoided	No other bias
1 LAN Jianqing	Yes	Unclear	Yes	Yes	Unclear	Yes	Yes
2 TAN Nian	Yes	Unclear	Yes	Yes	Unclear	Yes	Unclear
3 ZHANG Jianzhu	Unclear	Unclear	Yes	Yes	Unclear	Unclear	Unclear
4 CHEN Yongjun	No	Unclear	Yes	Yes	Unclear	Unclear	Yes
5 QIN Xufang	Unclear	Unclear	Yes	Yes	Unclear	Unclear	Yes
6 LI Baojiang	Unclear	Unclear	Yes	Yes	Unclear	Unclear	Yes
7 YAO Ke	Yes	Yes	Yes	Yes	Unclear	Yes	Yes
8 IZZET Can	Unclear	Unclear	Yes	Yes	Unclear	Unclear	Yes
9 JUN Wang	Unclear	Unclear	Yes	Yes	Unclear	Unclear	Yes
10 KEN Hayashi	Yes	Yes	Yes	Yes	Yes	Unclear	Yes
11 LIXIAO Luo	Yes	Yes	Yes	Yes	Yes	Unclear	Yes

## Results

### Selection and description of studies

**Fig. 1:**
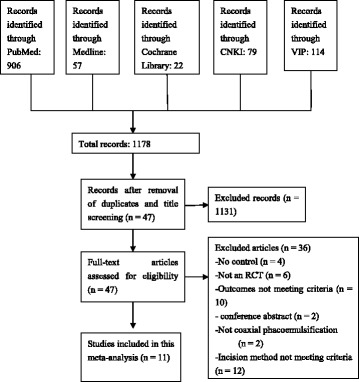
Flow chart of study selection

Initially, a total of 1178 records (193 in Chinese and 985 in English) were identified through the database search. After screening the titles, 1131 records were excluded because they were duplicates or unrelated to this meta-analysis. The full text of the 47 remaining records was assessed. After the completion of screening, 11 RCT studies [9–18] were included in this meta-analysis; 36 records were excluded because they described studies that were not randomized, lacked a control group, or did not report the outcomes of interest for this study. The flow chart of study selection are shown in Fig. 1. The selected studies included a total of 550 eyes in the MICS arms and 548 eyes in the SICS arms. The characteristics of the included studies are listed in Table 1.

### Risk of bias

**Fig. 2:**
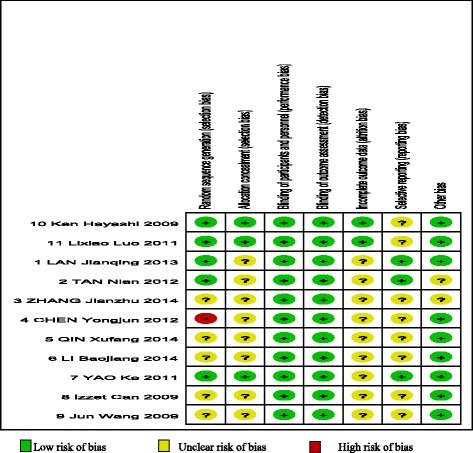
Risk of bias summary

**Fig. 3:**
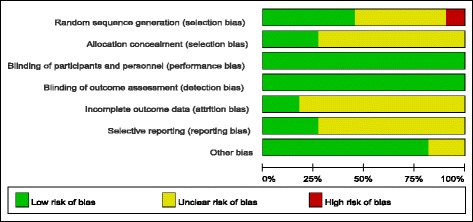
Risk of bias graph (We could not see the picture of Fig. 3.)

The results of the risk of bias assessment for the 11 included studies are shown in Figs. 2 and 3. Sequence generation was appropriate in five studies. While, one study was assessed a high risk on sequence generation for the random allocation was carried out according to registration order. Allocation concealment was described in three studies. Yet in the other studies, it was unclear. The outcomes involved in this meta-analysis were objective, which contributed to the low risk of bias associated with blinding of participants and personnel and blinding of outcome assessments. The outcomes data were complete in two studies, and other studies were unclear. Two studies avoided selective reporting, and others were unclear.

### Meta-analysis results

#### Surgically induced astigmatism

**Fig. 4:**
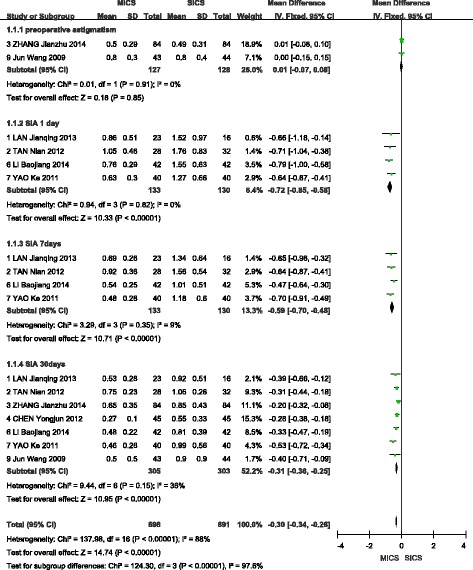
Forest plot of the SIA comparison (1 day, 7 days, 30 days postoperatively)

**Fig. 5:**
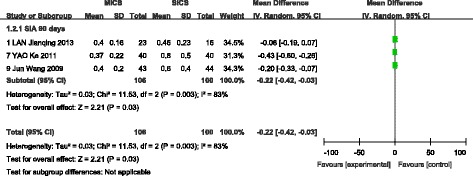
Forest plot of the SIA comparison (30 days postoperatively)

A fixed-effects model was selected when analysing SIA at 1 day, 7 days, and 30 days after surgery, as no significant heterogeneity was found among the included studies. A random-effects model was selected to analyse the outcomes of SIA at 90 days postoperatively due to significant heterogeneity among the included studies(I^2^ = 83%, *P* = 0.003). A total of two studies [13, 16] reported the prevalence of preoperative astigmatism, and no statistically significant difference was observed between MICS and SICS (WMD = 0.01, 95% CI (−0.07, 0.08), Z = 0.18, *P* > 0.05). A total of four studies [9, 11, 12, 15] reported outcomes of SIA at 1 day after surgery and showed less SIA with MICS than SICS (WMD = −0.72, 95% CI (−0.85, −0.58), Z = 10.33, *P* < 0.00001). A total of four studies [9, 11, 12, 15] reported outcomes of SIA at 7 days after surgery and again showed less SIA with MICS than SICS (WMD = −0.59, 95% CI (−0.70, −0.48), Z = 10.71, P < 0.00001). A total of seven studies [9–13, 15, 16] reported outcomes of SIA at 30 days after surgery and showed less SIA with MICS than SICS (WMD = −0.31, 95% CI (−0.36, −0.25), Z = 10.95, P < 0.00001). Finally, a total of three studies [9, 11, 16] reported outcomes of SIA at 90 days after surgery and indicated that MICS is superior to SICS (WMD = −0.22, 95% CI (−0.42, −0.03), Z = 2.21, *P* < 0.05). These results are shown in Figs 4 and 5.

### Effective phacoemulsification time

**Fig. 6:**
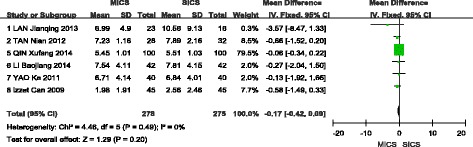
Forest plot of the EPT comparison

A total of six studies were included in the meta-analysis of this outcome [9, 11, 12, 14, 15]. A fixed-effects model was selected, as no significant heterogeneity was found among the studies (I^2^ = 0%, *P* = 0.49). The results indicated no statistically significant difference between MICS and SICS for the EPT (WMD = −0.17, 95% CI (−0.42, 0.09), Z = 1.29, *P* > 0.05), as shown in Fig. 6.

### Average ultrasound power

**Fig. 7:**
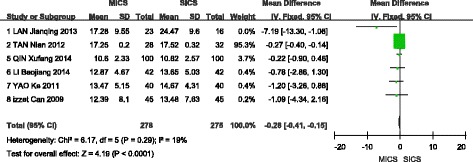
Forest plot of the AVE comparison

A total of six studies were included in the meta-analysis of this outcome [9, 11, 12, 14, 15]. A fixed-effects model was selected, as no significant heterogeneity was found among the studies (I^2^ = 19%, *P* = 0.29). The analysis showed a statistically significant difference in AVE between MICS and SICS (WMD = −0.28, 95% CI (−0.41, −0.15), Z = 4.19, *P* < 0.0001), as shown in Fig. 7.

### Central corneal thickness

**Fig. 8:**
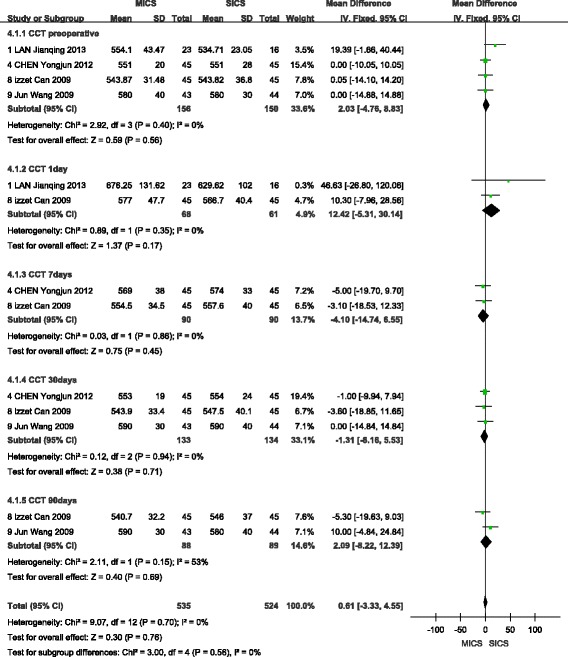
Forest plot of the CCT comparison

A fixed-effects model was selected to analyse this outcome. A total of four studies were included [9, 10, 16] in the analysis of preoperative CCT, which showed no statistically significant difference between MICS and SICS (WMD = 2.03, 95% CI (−4.76, 8.83), Z = 0.59, *P* > 0.05). A total of two studies [9] were included in the analysis of CCT at 1 day after surgery and found no statistically significant difference between MICS and SICS (WMD = 12.42, 95% CI (−6.31,30.14), Z = 1.37, P > 0.05). Similarly, the two studies [10] that reported outcomes of CCT at 7 days after surgery showed no statistically significant difference between MICS and SICS (WMD = −4.10, 95% CI (−14.74, 6.55), Z = 0.75, *P* > 0.05). A total significant difference of three studies [10, 16] reported outcomes of CCT at 30 days after surgery and again showed no statistically difference between MICS and SICS (WMD = −1.31, 95% CI (−8.16, 5.53), Z = 0.38, *P* > 0.05). Finally, a total of two studies [16] reported outcomes of CCT at 90 days after surgery and indicated no statistically significant difference between MICS and SICS (WMD = 2.09, 95% CI (− 8.22, 12.39), Z = 0.40, *P* > 0.05). These results are shown in Fig. 8.

### Endothelial cell count

**Fig. 9:**
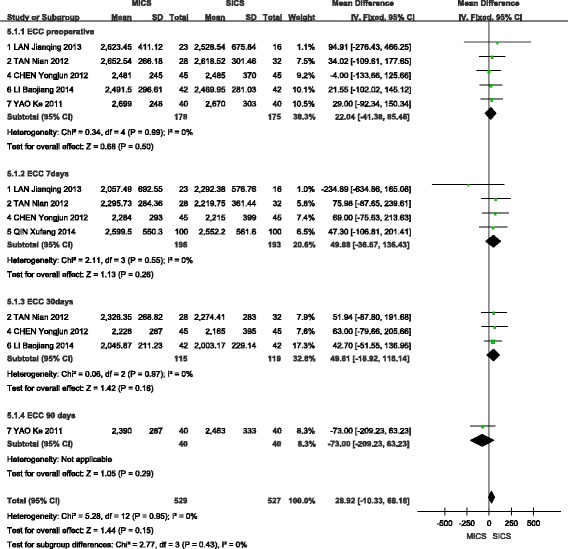
Forest plot of the ECC comparison

A fixed-effects model was selected to analyse this outcome, given the lack of heterogeneity among the included studies. A total of five studies [9–12, 15] were included in the analysis of preoperative ECC, which showed no statistically significant difference between MICS and SICS (WMD = 22.04, 95% CI (−41.38, 85.46), Z = 0.68, *P* > 0.05). A total of four studies [9, 10, 12, 14] reported outcomes of ECC at 7 days after surgery and showed no statistically significant difference between MICS and SICS (WMD = 49.88, 95% CI (−36.67, 136.34), Z = 1.13, *P* > 0.05). Finally, a total of three studies [10, 12, 15] reported outcomes of ECC at 30 days after surgery and again showed no statistically significant difference between MICS and SICS (WMD = 49.61, 95% CI (−18.92, 118.14), Z = 1.42, *P* > 0.05). These results are shown in Fig. 9.

### Endothelial cell count loss

**Fig. 10:**
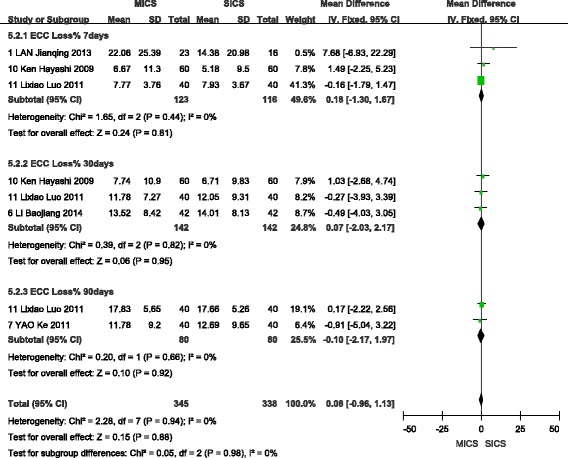
Forest plot of ECC Loss % comparison

A fixed-effects model analysis was selected to analyse this outcome, given the lack of heterogeneity observed among the included studies. A total of three studies [9, 17, 18] were included in the analysis of ECC Loss % at 7 days after surgery, which showed no statistically significant difference between MICS and SICS (WMD = 0.18, 95% CI (−1.30, 1.67), Z = 0.24, P > 0.05). Similarly, the three studies [15, 17, 18] that reported ECC Loss % at 30 days after surgery showed no statistically significant difference between MICS and SICS (WMD = 0.07, 95% CI (-2.03, 2.17), Z = 0.06, P > 0.05). Finally, a total of two studies [11, 18] reported ECC Loss % at 90 days after surgery and showed no statistically significant difference between MICS and SICS (WMD = −0.10, 95% CI (−2.17, 1.97), Z = 0.10, P > 0.05). These results are shown in Fig. 10.

### Publication bias analysis

**Fig. 11:**
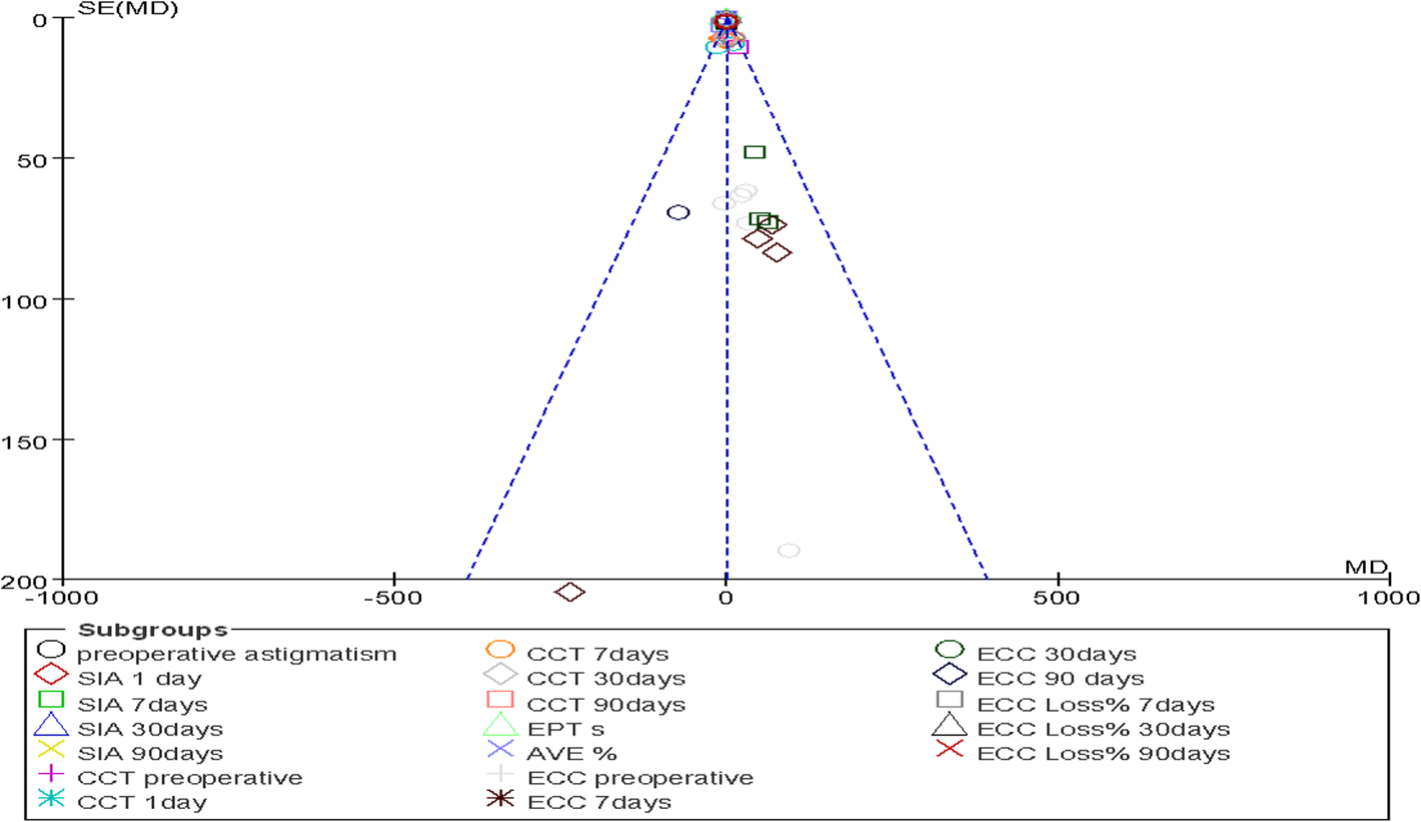
Funnel plot of comparisons of MICS and SICS

As shown in Fig. 11, most of the data bias was within the 95% CI, and this range included the null (zero). The distribution of the plots was symmetric. These results demonstrate that publication bias had no influence on the credibility of this research.

### Intraoperative and postoperative complications

The comparisons of complications between MICS and SICS are shown in Table 1. Corneal wrinkle, anterior chamber collapse, posterior capsule rupture, and iris prolapsed through the incision are the commonly reported intraoperative complications. Corneal edema is the commonly reported postoperative complications. All the complications showed no statistically significant differences between MICS and SICS.

## Discussion

Due to the rapid development of phacoemulsification and the trend towards minimally invasive surgery, incision size has begun to play a critical role in cataract surgery. Smaller incisions heal more rapidly and result in improved stability and impermeability of the anterior chamber, as well as less SIA and faster recovery of visual quality [6, 17, 19, 20]. However, research findings regarding the difference between MICS and SICS for the treatment of age-related cataracts are inconsistent. Some scholars suggest that MICS effectively reduces the AVE [9], ECC Loss % [10], corneal oedema [10] and SIA in both the short term and the long term compared to SICS [11]. However, some studies show no significant difference between MICS and SICS in the AVE [11], ECC Loss % [11], corneal oedema [11] and SIA in the long term [9]. These inconsistent conclusions create confusion for readers and clinicians. A previous study by Shentu and colleagues compared MICS with SICS, but it only evaluated outcomes up to 60 days after surgery. Comparisons of postoperative SIA only included short-term outcomes. Thus, a need remains for studies comparing long-term postoperative outcomes of MICS and SICS. Furthermore, difficulties in conducting clinical RCTs and limitations in sample size exacerbate differences among studies due to random error. To provide credible and conclusive evidence to readers, this study used meta-analysis methods, which can overcome the limitations of traditional clinical RCTs. A total of 11 studies were included in this meta-analysis. The current study is representative, as it involves studies conducted in several regions, and outcomes of SIA, EPT, AVE, CCT, ECC, ECC Loss %, intraoperative complications, and postoperative complications were selected to evaluate the effects of surgery. All selected outcomes are objective, and the risk of bias was low for all outcomes.

The results of this meta-analysis showed no statistically significant difference between MICS and SICS with regard to EPT, indicating that the duration of surgery does not differ between MICS and SICS. However, the AVE was significantly different between MICS and SICS. The surgical instruments used in MICS are more delicate, leading to lower AVE than in SICS.

The size and placement of the incision affect corneal curvature and SIA [21], which is the key factor that influences postoperative visual acuity [9]. Hayashi’s study showed that decreasing the size of the incision by 0.5 mm leads to a 0.25 D decrease in SIA [22]. The results of our study show a significant difference in SIA at 1, 7, 30, and 90 days postoperatively, indicating that MICS causes less SIA than SICS in both the short term and the long term. We therefore conclude that smaller incisions decrease SIA, which is consistent with the results of Kahraman and other authors [3–5]. What’s more, it is an important evidence that MICS has more superiority at a low SIA and better visual quality than SICS.

Corneal oedema is common after cataract surgery, and it can affect postoperative visual acuity and quality [11] by reducing the transparency of the cornea. The CCT reflects the degree of postoperative corneal oedema. The results of this study show no statistically significant difference in CCT at 1 7, 30, or 90 days postoperatively, indicating that MICS is similar to SICS in its effects on postoperative corneal oedema. Corneal endothelial cells play an important role in normal physiological function, and they are crucial for maintaining the transparency of the cornea [11]. The duration and energy level of ultrasound exposure, as well as the infusion of viscoelastic agents into the anterior chamber during surgery, can damage endothelial cells [15, 23]. This meta-analysis shows no significant difference in the ECC at 7 and 30 days, nor any difference in ECC Loss % at 7, 30, and 90 days postoperatively. Theoretically, MICS could hasten the closure of the anterior chamber [18] and decrease endothelial cell damage and loss [7]. However, the results of this study show no significant difference between MICS and SICS. Thermal damage associated with surgical instruments and larger energy consumption may contribute to ECC Loss %; the specific mechanisms involved require further research. CCT, ECC, and ECC Loss % are associated with corneal oedema and corneal function, which affect visual recovery and surgery safety. Thereby, the results provide evidence that MICS and SICS is similar in surgery safety.

The intraoperative and postoperative complications play important roles in evaluating the safety of cataract surgery. The commonly reported complications were corneal wrinkle, anterior chamber collapse, posterior capsule rupture, iris prolapsed through the incision, and corneal edema. As reported in the included studies, the corneal edema could be resolved by treatment. No s statistically significant differences between MICS and SICS on complications indicates that MICS is similar to SICS on surgery safety.

Endophthalmitis [24] and macular thickness [25] outcomes are also related to incision size. However, we did not analyse these outcomes because the number of relevant RCT reports is limited. The detailed mechanisms underlying the differences in MICS and SICS deserve further research; health economics evaluations of these treatments are also needed.

There were some limitations in this meta-analysis. First, some of the included studies provided no details on the method of randomization, allocation of concealment, or the intention-to-treat (ITT) analysis of patients who were lost to follow-up. The unclear risk of bias in these studies may affect the credibility of the results. Second, the presence of significant heterogeneity between included studies influences the credibility of the results. A sensitivity analysis was used to assess the robustness of the meta-analysis results and to analyze the source of heterogeneity by sequentially omitting individual studies. However, the sensitivity analysis is not suitable for SIA at 90 days postoperatively and CCT at 90 days postoperatively. The heterogeneity is obvious among studies included in analysing SIA at 90 days postoperatively (I^2^ = 83%, *P* = 0.003). The sensitivity analysis could not ascertain the source of heterogeneity, and the meta-regression analysis could not be used because the number of included studies is too few. Thus, a random-effects model was used to analyze SIA at 90 days postoperatively. Regarding CCT at 90 days postoperatively, the heterogeneity was encountered (I2 = 53%, *P* = 0.15). A fixed-effects model was selected for the heterogeneity show no statistical differences (P = 0.15) and the number of included studies limits further analysis. Additionally, the statistical results of the fixed-effects model are consistent with that of the random-effects model on SIA at 90 days postoperatively and on CCT at 90 days postoperatively. The detection of heterogeneity is related to the diversity of clinical characteristics which affect the uniformity of the involved studies. For example, the patients maybe have different ages or come from different regions and races. Plus, the surgeries maybe were conducted by different doctors using different equipments in each included study. Third, as we could not gain access to unpublished results, publication bias cannot be fully excluded.

## Conclusion

The results of this meta-analyses show that MICS has more superiorities than SICS and that the switching from SICS to MICS is reasonable. Compared to SICS, MICS can reduce short-term and long-term SIA but produces no difference in corneal oedema, endothelial cell loss, operation time, intraoperative complications, or postoperative complications. The surgery safety of MICS is similar to that of SICS. Therefore, MICS has more advantages than SICS in reducing SIA. We would like to recommend the clinicians to promote MICS. Higher-quality randomized controlled studies are needed to validate these findings.

## Additional file

Additional file 1:
**Table S1.** Search strategy for PubMed. (DOC 31 kb)

## Abbreviations

AVE: Average ultrasonic energy
CCT: Central corneal thickness
CNKI: National Knowledge Infrastructure
ECC Loss %: Endothelial cell count percentage
ECC: Endothelial cell count
EPT: Effective phacoemulsification time
ITT: Intention-to-treat
MICS: Microincisional phacoemulsification surgery
RCT: Randomized controlled trial
SIA: Surgically induced astigmatism
SICS: Standard-incision phacoemulsification surgery

## References

[1] Lou S, Yuan Y (2012). Epidemiology investigation of the senile cataract. Journal of nanchang university (medical edition).

[2] Zhao L, Yan H (2012). Advantages and disadvantages of microincision cataract surgery. Int J Ophthalmol..

[3] Can I, Takmaz T, Yildiz Y (2010). Coaxial, microcoaxial, and biaxial microincision cataract surgery: prospective comparative study. Cataract Refract Surg..

[4] Kahraman G, Amon M, Franz C (2007). Intraindividual comparison of surgical trauma after bimanual microincision and conventional small-incision coaxial phacoemulsification. Catarac Refract Surg.

[5] Kurz S, Krummenauer F, Gabriel P (2006). Biaxial microincision versus coaxial smallincision clear cornea cataract surgery. Ophthalmology.

[6] Dosso AA, Cottet L, Burgener ND, Di Nardo S (2008). Outcomes of coaxial microincision cataract surgery versus conventional cataract surgery. Cataract Refract Surg..

[7] Mencucci R, Ponchietti C, Virgili G, Giansanti F, Menchini U (2006). Corneal endothelial damage after cataract surgery: microincision versus standard technique. Cataract Refract Surg..

[8] Liu Y (2010). Update on Microincisional Phacoemulsification. Journal of Sun Yat-Sen University (Medical Sciences).

[9] Lan J, Guo H, Cui Y (2013). Comparative study on clinical effects of 1.8mm coaxial micro-incision and 3.0 mm standard incision in cataract phacoemulsification surgery. Recent Advin Ophthalmol.

[10] Chen Y, Su L, Tian F (2012). Clinical application of 2.2 mm micro-incision phacoemulsification in cataract surgery. Recent Adv Ophthalmol..

[11] Yao K, Wang W, Wu W (2011). Clinical evaluation on the coaxial 1.8 mm microincision cataract surgery. Chinese J Ophthalmol.

[12] Tan L, Ye J (2012). Phacoemulsification through 1.8 mm coaxial microincision. Third Military Medical University J.

[13] Zhang J, Chen J, Huang B (2014). Effect of 1.8mm coaxial micro-ncision cataract phacoemulsification on corneal astigmatism. Int J Ophthalmol.

[14] Qin X, Yang X, Quan C (2014). Comparative study of 2.2 mm coaxiaI micro incision phacoemulsification and traditional phacoemulsification in cataract operation. Chin J Mod Drug Appl.

[15] Li B (2014). Clinical evaluation on 2.2 mm micro-incision cataract surgery. Recent Advances in Ophthalmology..

[16] Wang J, Zhang E, Fan W (2009). The effect of micro-incision and small-incision coaxial phaco-emulsification on corneal astigmatism. Clin Exp Ophthalmol.

[17] Hayashi K, Yoshida M, Hayashi H (2009). Postoperative corneal shape changes: microincision versus smallincision coaxial cataract surgery. Cataract Refract Surg..

[18] Luo L, Lin H, He M (2012). Et a1.Clinical evaluation of three incision size–dependent Phacoemulsification systems. Am J Ophthalmol.

[19] Berdahl JP, DeStafeno JJ, Kim T (2007). Corneal wound architecture and integrity after phacoemulsification: evaluation of coaxial microincision coaxial,and microincision bimanual techniques. Cataract Refract Surg..

[20] Soscia W, Howard JG, Olson RJ (2002). Bimanual phacoemulsification through 2 stab incisions:a wound temperature study. Cataract Refract Surg..

[21] Zhong J, Shao D, Liu P (2004). Effect of different corneal incisions on corneal refraction after phacoemulsification and foldable posterior chamber intraocular lens implantation. Recent Adv Ophthalmol.

[22] Hayashi K, Hayashi H, Nakao F (1995). The correlation between incision size and corneal shape changes in sutureless cataract surgery. Ophthalmology.

[23] Milla E, Verges C, Cipres M (2005). Corneal endothelium evaluation after phacoemulsification with continuous anterior chamber infusion. Cornea.

[24] Masket S, Belani S (2007). Proper wound construction to prevent short-term ocular hypotony after clear corneal incision cataract surgery. Cataract Refract Surg.

[25] Ghosh SI, Roy I, Biswas PN (2010). Prospective randomized comparative study of macular thickness following phacoemulsification and manual small incision cataract surgery. Acta Ophthalmol.

