# Supplementary material for: Comparison of efficacy between coaxial microincision and standard-incision phacoemulsification in patients with age-related cataracts: a meta-analysis
Source: BMC Ophthalmol. 2017 Dec 29;17:267. doi: 10.1186/s12886-017-0661-6 (PMC5747124; doi:10.1186/s12886-017-0661-6)
Supplement: Additional file 1: — Table S1. Search strategy for PubMed. (DOC 31 kb) [file 12886_2017_661_MOESM1_ESM.doc]

S1 Table. Search strategy for PubMed

| #1 | cataract OR age related cataract OR senile cataract [Title/Abstract] |
| --- | --- |
| #2 | phacoemulsification OR ultrasonic emulsification for cataract [Title/Abstract]) |
| #3 | micro incision OR MICS OR incision [Title/Abstract] |
| #4 | standard incision OR SICS OR Incision [Title/Abstract]) |
| #5 | #3 OR #4 |
| #6 | #1 AND #2 AND #5 |

(((cataract[Title/Abstract] OR age related cataract[Title/Abstract] OR senile cataract[Title/Abstract])) AND (phacoemulsification[Title/Abstract] OR ultrasonic emulsification for cataract[Title/Abstract])) AND (micro incision[Title/Abstract] OR MICS[Title/Abstract] OR standard incision[Title/Abstract] OR SICS[Title/Abstract] OR Incision[Title/Abstract])
